# Comparison of Nitroglycerin-Induced Pressure Ratio Drop and Resting Full-Cycle Ratio in a Pressure Wire Study

**DOI:** 10.3390/jcm13226716

**Published:** 2024-11-08

**Authors:** Chien-Boon Jong, Tsui-Shan Lu, Min-Tsun Liao, Jia-Lang Xu, Chun-Kai Chen, Jui-Cheng Kuo, Chih-Cheng Wu

**Affiliations:** 1 Department of Internal Medicine, National Taiwan University Hospital, Hsin-Chu Branch, Hsin-Chu 300195, Taiwan; liaomintsun@gmail.com (M.-T.L.); ineosky@gmail.com (C.-K.C.); chihchengwumd@gmail.com (C.-C.W.); 2 College of Medicine, National Taiwan University, Taipei 100233, Taiwan; 3 Department of Mathematics, National Taiwan Normal University, Taipei 116059, Taiwan; tslu@ntnu.edu.tw; 4 Big Data Center, National Chung Hsing University, Taichung 402202, Taiwan; jlxu@nchu.edu.tw; 5 Department of Computer Science & Information Engineering, Chaoyang University of Technology, Taichung 413310, Taiwan; 6 Department of Radiology, National Taiwan University Hospital, Hsin-Chu Branch, Hsin-Chu 300195, Taiwan; lonelystaring@gmail.com

**Keywords:** fractional flow reserve, nitroglycerin, pressure ratio drop, resting full-cycle ratio

## Abstract

**Background/Objectives**: The acute drop in distal coronary pressure (Pd)-to-aortic pressure (Pa) ratio after intracoronary nitroglycerin (NTG-Pd/Pa) administration is an acceptable estimate of fractional flow reserve (FFR). We aimed to compare the diagnostic performance of NTG-Pd/Pa with that of the resting full-cycle ratio (RFR) in predicting the binary results of FFR. **Methods**: This study included two prospective studies registered under the numbers NCT04700397 and NCT03693157. Altogether, 202 vessels were included. The optimal cutoff of NTG-Pd/Pa for predicting FFR ≤ 0.8 was identified and validated in another prospective registry. We used the McNemar’s test and the DeLong method to compare the diagnostic efficiency of NTG-Pd/Pa vs. RFR in predicting FFR ≤ 0.8 in a pooled cohort. **Results**: NTG-Pd/Pa was strongly correlated with FFR (r = 0.945, *p* < 0.001). The NTG-Pd/Pa cutoff for predicting FFR ≤ 0.8 was 0.85 in both the derivation and validation cohorts. The area under the receiver-operating characteristic curve (AUC) and accuracy in predicting FFR ≤ 0.8 were higher for NTG-Pd/Pa than for RFR in the pooled cohort (AUC 0.97 vs. 0.91, *p* < 0.001; accuracy 91% vs. 84%, *p* < 0.001). The sensitivity and negative predictive values were also higher for NTG-Pd/Pa than for RFR (all *p* < 0.05). The specificity and positive predictive value were numerically higher for NTG-Pd/Pa than for RFR (all *p* > 0.05). **Conclusions:** The diagnostic performance of NTG-Pd/Pa may surpass that of the RFR in predicting the binary results of the FFR.

## 1. Introduction

Functional assessment of coronary artery stenosis using the fractional flow reserve (FFR) is performed under hyperemic conditions. Currently, the guidelines of myocardial revascularization recommend using the FFR to assess the hemodynamic relevance of intermediate-grade stenosis [1,2]. However, hyperemia induction with adenosine involves certain costs and risks [3]. Recently, several non-hyperemic pressure ratios (NHPRs) have been introduced to overcome these limitations and have demonstrated comparable clinical outcomes with FFR [4,5]. Currently, the measurement of these NHPRs is limited to the proprietary software of individual vendors, limiting their clinical application.

Intracoronary nitroglycerin (NTG) injection is mandatory for evaluating physiological ischemia of the myocardium by FFR or NHPR measurement. NTG causes coronary vasodilatation, which increases myocardial blood flow and decreases coronary vascular resistance, thereby mimicking the action of hyperemic agents [6]. The acute drop in the ratio of the mean arterial pressure distal (Pd) to the stenosis and mean aortic pressure at the tip of the guiding catheter (Pa) after intracoronary NTG injection (NTG-Pd/Pa) is an acceptable estimate of FFR [7]. However, NTG administration may elicit life-threatening hypotension [8], and a standardized dosing strategy may not be suitable in clinical practice.

In the first part of this study, we aimed to report the feasibility of pressure-based NTG administration in an NTG-Pd/Pa assessment and validate the relationship between NTG-Pd/Pa and FFR values in another prospective registry. We hypothesized that NTG dosage and NTG-Pd/Pa assessment timing would not alter the relationship between NTG-Pd/Pa and FFR. Second, we aimed to compare the diagnostic performance of NTG-Pd/Pa with that of the resting full-cycle ratio (RFR) in predicting the binary results of FFR.

## 2. Materials and Methods

### 2.1. The Study Population

**Figure 1:**
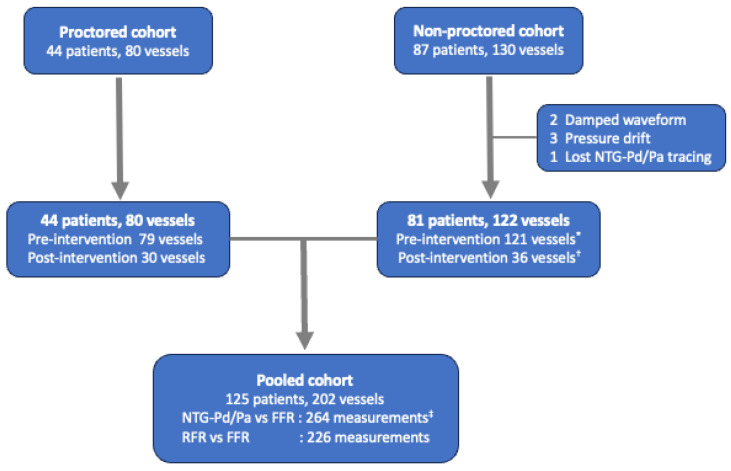
Diagram of patient flow. In the proctored cohort, 44 patients with 80 vessels are administered intracoronary NTG according to a blood pressure-based NTG dose adjustment protocol. Overall, 109 NTG-Pd/Pa measurements are performed, including 79 and 30 measurements assessed pre- and post-intervention, respectively. In contrast, the non-proctored (validation) cohort initially includes 87 patients with 130 vessels. After a quality check of the pressure tracing, six patients are excluded because of significant pressure drift (three patients), damped aortic waveform during NTG-Pd/Pa measurement (two patients), and loss of NTG-Pd/Pa value (one patient). In addition, another three vessels met the criteria of significant drift during the pre-intervention FFR assessment but passed the quality check in the post-intervention FFR assessment. Therefore, 81 patients with 157 NTG-Pd/Pa measurements are enrolled in the non-proctored cohort of the present study, including 121 and 36 measurements assessed pre- and post-intervention, respectively. The pooled cohort comprises 125 patients with 202 vessels. Overall, 264 measurements are included to evaluate the relationship between NTG-Pd/Pa and FFR, and 226 paired measurements are included to compare the diagnostic performance of NTG-Pd/Pa and RFR in predicting the binary results of the FFR. * Three vessels are excluded because of significant drift. ^†^ One vessel is excluded because of significant drift. ^‡^ Two vessels are excluded because of a lack of paired FFR assessment due to bradycardia. Abbreviations: FFR, fractional flow reserve; NTG, nitroglycerin; NTG-Pd/Pa, nitroglycerin-induced acute drop in Pd/Pa; Pa, aortic pressure; Pd, distal coronary pressure; RFR, resting full-cycle ratio.

This study included two prospective studies conducted at our institute (registered under the numbers NCT04700397 and NCT03693157). In the first part of the study, we enrolled patients who had intermediate lesions with 30–90% stenosis (by visual estimation) in coronary artery diameter ≥ 2.5 mm and intended to undergo FFR assessment [1,9]. NTG-Pd/Pa was assessed according to the study protocol, and the reproducibility of the NTG-Pd/Pa test was assessed after the FFR examination at the operator’s discretion (NCT04700397). Subsequently, we validated the relationship between NTG-Pd/Pa and the FFR using another recent prospective registry, wherein the efficacy and safety of high-dose intracoronary adenosine escalation were evaluated during initial FFR assessments [10] (NCT03693157). Both prospective studies had similar inclusion criteria, and patients with severe aortic valve stenosis, adenosine hypersensitivity history, resting heart rate of <50 beats per minute, systolic blood pressure of <90 mmHg, or asthma history were excluded. In the second part of the study, we pooled these two prospective studies to evaluate the diagnostic performance of NTG-Pd/Pa and RFR separately, using FFR ≤ 0.80 as the ischemic reference (Figure 1). Both prospective studies recommended an RFR assessment before the FFR examination, although it was not compulsory. The present study was conducted in accordance with the Declaration of Helsinki and relevant regulations and approved by the Institutional Review Board of the National Taiwan University Hospital Hsin-Chu Branch (IRB No. 109-081-E and 111-202-E). All participants provided written informed consent.

### 2.2. Study Protocol

After standard preparation, the pressure guidewire with a pressure sensor at the tip was introduced into a 6- or 7-gauge French guiding catheter. The Pa and Pd were equalized at the ostium of the left or right coronary artery at the beginning of the procedure [11]. Subsequently, the pressure sensor was introduced into the distal third of the target vessel. The two commercial pressure guidewires used in these studies were the PressureWire X (Abbott Cardiovascular, St. Paul, MN, USA) and COMET II (Boston Scientific, Marlborough, MA, USA). First, the NTG-Pd/Pa was measured after intracoronary NTG administration [10]. A protocol was designed with NTG dosage adjustment based on the instantaneous blood pressure, and this blood pressure-based adjustment was also applied in the reproducibility test of NTG-Pd/Pa (Supplementary Figure S1). Patients with a systolic blood pressure of <90 mmHg were excluded from the present study to avoid further hypotension after NTG administration. Second, the RFR, or diastolic hyperemia-free ratio (the diastolic hyperemia-free ratio was excluded from this study), was measured after the Pd/Pa ratio was stabilized. Third, the FFR was obtained after inducing hyperemia via intracoronary adenosine administration. In the derivation cohort, 200 μg and 100 μg of adenosine were administered to the left and right coronary arteries, respectively. Adenosine up-titration to doses of 400–600 μg and 200 μg in each coronary artery was recommended when the first FFR value was obtained in the 0.76–0.85 range. In contrast, the high-dose adenosine escalation strategy was used in the validation cohort. The intracoronary adenosine doses were gradually increased from 100 μg to 200, 300, 400, 600, and 800 μg during the FFR assessment, according to the study protocol (Supplementary Figure S2). This escalation protocol was immediately terminated in case of FFR decrease to ≤0.75, bradycardia (a complete atrioventricular block or a long pause of >3 s), intolerable chest discomfort, or minimum Pd/Pa was achieved [10]. Finally, the pullback and final pressure tracings were recorded to check the pressure drift at the end of the procedure (Supplementary Figures S1 and S2). The procedure was repeated if the pressure drift exceeded 0.03. All physiological indices were excluded from analysis in the present study if the final pressure drift (Pd/Pa ratio) exceeded 1 ± 0.03 [11]. Otherwise, the NTG-Pd/Pa in the validation cohort (NCT03693157) was commonly measured after administering 200 μg of NTG and without proctoring, which was recommended in the abovementioned study. However, doses of 100 μg were also acceptable in the abovementioned study at the discretion of the operator [10]. The cohort that underwent NTG-Pd/Pa measurement with pressure-based NTG dosage adjustment was termed the proctored cohort, and the cohort that underwent an NTG-Pd/Pa measurement without proctoring was the non-proctored cohort (Figure 1).

### 2.3. Data Acquisition and Quality Check (NTG-Pd/Pa, FFR, and RFR)

All data regarding pressure tracings were recorded, and offline analysis was performed on the console of the FFR system (QUANTIEN Measurement System; v.1.12, 2015, Abbott Cardiovascular, St. Paul, MN, USA; POLARIS Multi-Modality Guidance System; v.2.1, 2019, Boston Scientific Corporation, San Jose, CA, USA) and a customized software program.

A physiology team comprising an experienced cardiologist (C.B.J.) and two trained technicians (R.C.K. and J.L.X.) determined the pressure waveform and value of the physiological index, with the assistance of an artificial intelligence-based software program. The pressure drift was rechecked offline before the analysis of each physiological index, and only data within an acceptable drift range were used in the present study. An artificial intelligence-based software program was used to check for pressure damping, waveform distortion, and artifact interruption. The NTG-Pd/Pa value was acquired at the lowest Pd/Pa after NTG administration, avoiding measurements in the first three pressure waveforms and premature capture beats (Supplementary Figure S3). FFR and RFR were calculated using the methods described in previous reports [10]. Briefly, the FFR was defined as the lowest Pd/Pa after intracoronary adenosine administration, while disregarding measurements obtained in the first three pressure waveforms or during ectopic heartbeats. The RFR was defined as the lowest Pd/Pa ratio during the entire cardiac cycle [12]. The physiology team was blinded to the clinical data, coronary angiography results, and results of other physiological indices.

### 2.4. Statistical Analysis

The baseline characteristics of the pooled cohort and two classified cohorts were tabulated. Pearson’s linear correlation test was used to determine the strength of the linear association between NTG-Pd/Pa and FFR. Receiver-operating characteristic (ROC) curves were plotted to summarize the classifier performance of NTG-Pd/Pa and identify the optimal cutoff thresholds of NTG-Pd/Pa to predict ischemia (defined as FFR ≤ 0.80), based on the method of Youden’s index [13]. The Wilcoxon signed-rank test was used to evaluate the difference between the reproducibility test of the NTG-Pd/Pa measurement. To evaluate the performance of the diagnostic tests of NTG-Pd/Pa (using the aforementioned cutoff) and RFR to predict the binary results of ischemia, the McNemar’s test was used to compare the differences in sensitivity, specificity, and accuracy, thereby accounting for the nature of dependence between the two tests. The generalized score statistic was used to compare the differences in the positive predictive value (PPV) and negative predictive value (NPV) [14]. The areas under the ROC curves (AUCs) were compared and tested through the DeLong method [15]. All statistical analyses were performed using the Statistical Analysis Software (SAS, version 9.4, SAS Institute Inc., Cary, NC, USA). The statistical significance was set at *p* < 0.05.

## 3. Results

**Figure 2:**
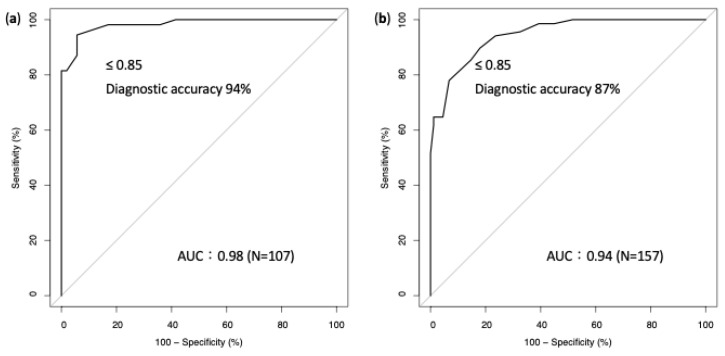
Receiver-operating characteristic curve and optimal cutoff of NTG-Pd/Pa for predicting a positive FFR. (**a**) Proctored and (**b**) non-proctored cohorts. The area under the curve of NTG-Pd/Pa for predicting a positive FFR is high in both cohorts, and the ischemic cutoff for NTG-Pd/Pa is identical in both cohorts. Abbreviations: FFR, fractional flow reserve; NTG-Pd/Pa, nitroglycerin-induced acute drop in Pd/Pa; Pa, aortic pressure; Pd, distal coronary pressure.

In the proctored cohort, the mean age was 65 years, and 82% were men. Half of the patients had diabetes, and nearly half had chronic kidney disease. One-quarter of current smokers and 15.9% of the patients had prior myocardial infarction. One of the six patients presented with acute coronary syndrome, and 17.5% and 42.5% of the target vessels were the right coronary artery and left anterior descending artery, respectively (Supplementary Table S1). Nearly one-third of the target lesions were located at the ostium or proximal part of the epicardial vessels. In most of the lesions, stenosis was in the 50–70% range, and 55.8% of pre-intervention FFR was ≤0.80. The median values of FFR, RFR, and NTG-Pd/Pa at the pre-intervention were 0.79, 0.91, and 0.85, respectively. In the proctored cohort, 109 NTG-Pd/Pa values were measured, and 200 μg of NTG was used in most of the measurements (85.3%). Two patients exhibited the Bezold–Jarish reflex (BJR) after NTG administration [8] (one with 100 μg of NTG in the left circumflex artery and another with 200 μg of NTG in the left anterior descending artery). Both BJRs resolved after hydration with a large bolus of saline and intravenous atropine administration. Five patients underwent repeat NTG-Pd/Pa measurements of the same lesions at least 4 min apart (three lesions in the left coronary artery and two in the right coronary artery). The NTG-Pd/Pa measurement demonstrated good reproducibility (*p* = 0.75). The Pearson’s linear relationship between NTG-Pd/Pa and FFR was good (r = 0.963). The AUC for predicting myocardial ischemia was 0.98, and the ischemic cutoff was 0.85 (Figure 2).

In the validation cohort (non-proctored), the mean age was 67 years, and 75% were men. Nearly half of the patients had diabetes, and 33% had chronic kidney disease. One-fourth of the patients presented with acute coronary syndrome, and 24.6% and 50.8% of the target vessels were in the right coronary artery and left anterior descending artery, respectively. One-third of target lesions were located at the ostium or proximal part of the epicardial vessels. In most of the lesions, stenosis was in the 50–70% range, and 47.1% of pre-intervention FFR was ≤0.80. The median values of FFR, RFR, and NTG-Pd/Pa at pre-intervention were 0.82, 0.92, and 0.87, respectively. Overall, 157 NTG-Pd/Pa values were measured, and 75% of these measurements were performed after administration of 200 μg of NTG (Supplementary Table S1). The Pearson’s linear relationship between NTG-Pd/Pa and FFR was good (r = 0.922). The AUC for predicting myocardial ischemia was 0.94, and the ischemic cutoff was identical to that of the proctored cohort (0.85) (Figure 2).

**Figure 3:**
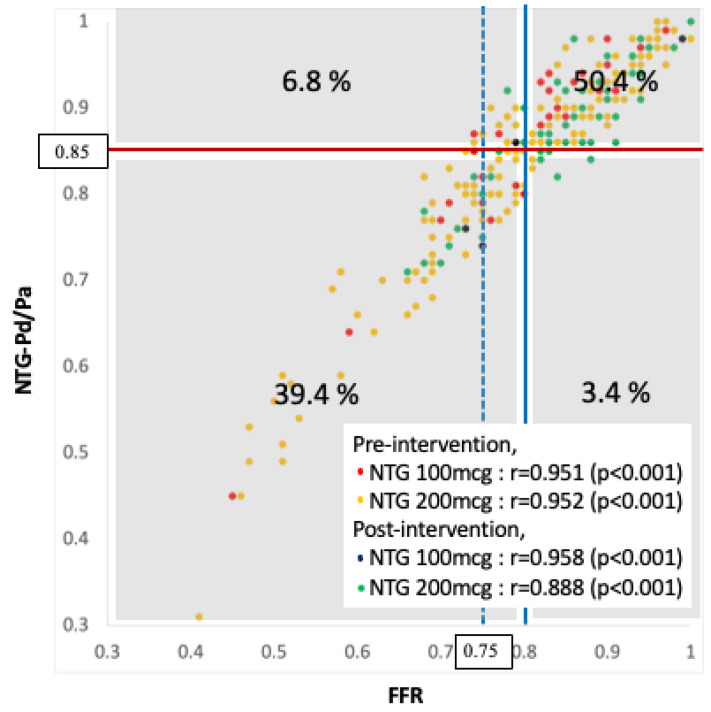
Scatter plot of the NTG-Pd/Pa vs. FFR in the pooled cohort. The relationship between NTG-Pd/Pa and FFR is good (r = 0.945 in the pooled cohort), irrespective of the pre- or post-intervention measurement and NTG administration (100 or 200 μg). Using a cutoff of 0.80 (blue line) and 0.85 (red line) for FFR and NTG-Pd/Pa, respectively, one-tenth of the results are discordant. However, <1% of the target vessels are false negative when using the 0.85 cutoff for NTG-Pd/Pa to predict the truly ischemic cutoff (FFR < 0.75). Abbreviations: FFR, fractional flow reserve; NTG, nitroglycerin; NTG-Pd/Pa, nitroglycerin-induced acute drop in Pd/Pa; Pa, aortic pressure; Pd, distal coronary pressure.

In the pooled cohort, 198 vessels underwent pre-intervention invasive physiological tests, and 66 received post-intervention tests. One-fifth of the vessels received 100 μg of NTG, and 80% of the vessels received 200 μg of NTG (Supplementary Table S1). The relationship between NTG-Pd/Pa and FFR was good (overall r = 0.945), irrespective of the pre- or post-intervention measurement and NTG administration (100 or 200 μg) (Figure 3). The AUCs of the pooled cohort, vessels that received 100 and 200 μg of NTG, were 0.96, 0.98, and 0.96, respectively (Supplementary Figure S4). The ischemic cutoff value of the pooled cohort was also 0.85.

**Figure 4:**
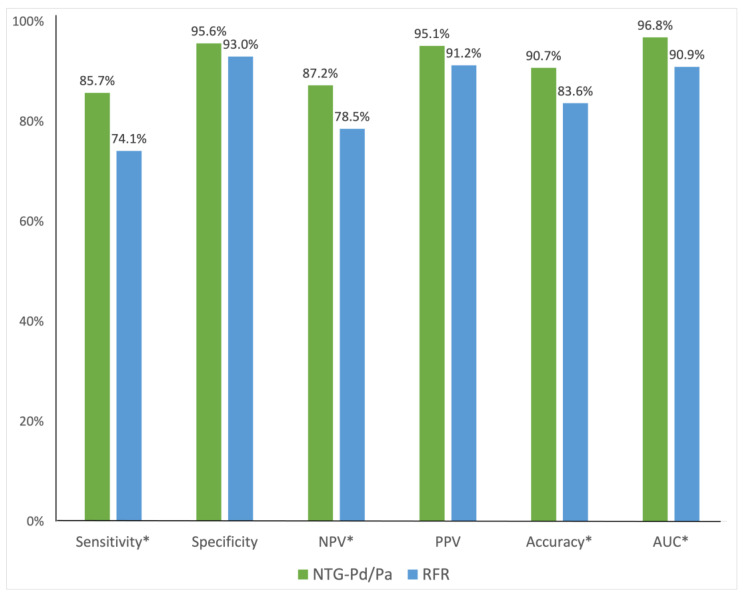
Diagnostic performance of NTG-Pd/Pa and RFR in predicting a positive FFR. The sensitivity, NPV, and diagnostic accuracy of NTG-Pd/Pa for predicting a positive FFR are higher than those of the RFR. However, the specificity and PPV are high for both NTG-Pd/Pa and RFR, without a statistically significant difference. * *p* < 0.05. Abbreviations: AUC: area under the receiver-operating characteristic curve; NPV, negative predictive value; NTG-Pd/Pa, nitroglycerin-induced acute drop in the ratio of distal coronary pressure to aortic pressure; Pa, aortic pressure; Pd, distal coronary pressure; PPV, positive predictive value; RFR, resting full-cycle ratio.

Next, 226 paired physiological indices (prediction of NTG-Pd/Pa for myocardial ischemia vs. prediction of RFR for myocardial ischemia) were analyzed. Among the paired physiological indices, 177 pairs were assessed at pre-intervention and 49 at post-intervention. Overall, the diagnostic accuracy, sensitivity, and NPV of NTG-Pd/Pa in predicting myocardial ischemia were higher than those of RFR (accuracy: 91% vs. 84%, *p* < 0.001; sensitivity: 86% vs. 74%, *p* = 0.007; NPV: 87% vs. 79%, *p* < 0.001). However, the specificity and PPV were high for both NTG-Pd/Pa and RFR, with no statistically significant differences (specificity: 96% vs. 93%, *p* = 0.257; PPV: 95% vs. 91%, *p* = 0.149). In addition, the AUC of NTG-Pd/Pa for predicting ischemia was higher than that of RFR (0.97 vs. 0.91, *p* < 0.001) (Figure 4).

## 4. Discussion

This study reported the feasibility of blood pressure-based NTG administration in NTG-Pd/Pa assessments. The NTG-Pd/Pa was closely correlated with the FFR in both the derivation and validation cohorts. Similarly, the cutoff value of NTG-Pd/Pa for predicting FFR ≤ 0.8 was identical in both cohorts, which recruited participants during different years. Furthermore, the different NTG doses (100 vs. 200 μg) and the different timings of the NTG-Pd/Pa assessment (pre- vs. post-intervention) did not alter the relationship between NTG-Pd/Pa and FFR. Therefore, NTG-Pd/Pa may be considered a novel index for the physiological assessment of coronary stenosis. To the best of our knowledge, this is the first study to show that the diagnostic performance of NTG-Pd/Pa may be better than that of the RFR in predicting binary results of the FFR.

Martin-Reyes showed a good correlation between NTG-Pd/Pa and FFR in the CANICA (Can Intracoronary Nitroglycerin Predict Fractional Flow Reserve Without Adenosine?) multicenter study [7]. A standard 200 μg dose of NTG was administered intracoronary in the CANICA study without considering the limitation of low blood pressure before the NTG injection. However, no procedure-related complications were reported during NTG-Pd/Pa measurements. In contrast, two cases of NTG-elicited BJR were recorded during the post-intervention physiological assessment in our proctored group, although blood pressure-based NTG administration had already been performed. Notably, both events occurred in the left coronary artery, which had previously received similar doses of intracoronary NTG in the pre-intervention FFR assessment, without triggering the BJR. This life-threatening phenomenon appears inconsistent and may change over time [8,16]. To date, there are no predictors of this phenomenon, and its mechanism remains unclear [16,17]. Therefore, careful monitoring and prevention of exaggerated hypotension are crucial during NTG administration. Currently, intracoronary administration of 200–300 μg of NTG before pressure wire assessment is recommended to prevent coronary spasms. Otherwise, the spasm may not be relieved if NTG underdosing leads to false positives during the pressure wire assessment. Therefore, a protocol involving an appropriate and safe dose of NTG is required for coronary procedures. The feasibility and safety of our study protocol were demonstrated in the present study, and our results may apply to other coronary procedures. In addition, the result of the NTG-Pd/Pa assessment with the administration of 100 μg of NTG was comparable to the administration of 200 μg of NTG in the present study. These results address the gap regarding dose-independent responses in the NTG-Pd/Pa assessment.

A few studies have shown that NTG can induce partial hyperemia in the coronary arteries. Bernstein et al. reported that intracoronary NTG administration increased the myocardial blood flow and reduced coronary vascular resistance in both normal and diseased coronary arteries in males [6]. In addition, Egashira et al. reported that NTG administration increased coronary blood flow equivalently in patients with or without microvascular dysfunction [18]. Habazettl et al. showed that the administration of either adenosine or NTG in dogs could induce vasodilatation of coronary microvessels with diameters ranging between 20 and 500 μm, as observed using intravital fluorescence microscopy. In that animal experiment, intracoronary infusion of adenosine and NTG equally achieved the near-maximum dilatation in coronary arterioles >100 μm. However, in arterioles <100 μm, the dilatation effect was lower after NTG administration than after adenosine administration. This difference markedly lowered microcirculatory resistance and maximized the coronary flow during adenosine infusion [19]. To date, several human studies have shown the efficacy of NTG administration in coronary blood flow augmentation and microcirculatory resistance reduction when evaluated via contemporary invasive hemodynamic measurement [20,21]. Therefore, NTG-Pd/Pa represented “partial hyperemia” induced by intracoronary NTG administration in this study. In contrast, the RFR is an NHPR, defined as the lowest filtered instantaneous Pd/Pa within the whole cardiac cycle [12]. The RFR is the diagnostic equivalent of the well-known instantaneous wave-free ratio and has been validated in contemporary practice [22,23]. The NHPR was measured with the assumption that the maximal flow and minimal resistance occurred in the “wave-free” period in the non-hyperemic condition, and the Pd/Pa measured within this period was closely correlated with the FFR result [24]. However, NHPR was not fully identical to FFR, and a discordance of approximately 20% was commonly reported in the literature [23,25,26,27,28]. We showed that the diagnostic performance of NTG-Pd/Pa surpassed that of RFR in predicting the binary results of FFR. Unsurprisingly, this result can be interpreted as the partial hyperemic index being logically better than the non-hyperemic index in predicting the maximal hyperemic index.

Intracoronary NTG administration is mandatory before a pressure wire study to prevent coronary spasms. This study demonstrated the feasibility of NTG-Pd/Pa for the physiological assessment of coronary artery stenosis. After reviewing the reported physiological indices in the literature, NTG-Pd/Pa had the highest AUC, diagnostic accuracy, and sensitivity for predicting FFR ≤ 0.8 while preserving the high specificity [12,25,29] (Supplementary Table S2). A less expensive and simplified procedure may be achieved using this novel index. Furthermore, the assessment of NTG-Pd/Pa was available from all vendors. This is particularly useful for patients with contraindications for adenosine use, such as those at risk for sick sinus syndrome and high-degree atrioventricular block. As opposed to contrast-based FFR, NTG-Pd/Pa is a novel partial hyperemic physiological index that lowers the risk of contrast toxicity [30].

This study had several limitations. First, the reproducibility test of the NTG-Pd/Pa measurements involved a small sample size. Repeated measurements of the NTG-Pd/Pa value within the same lesion were initially encouraged. However, the operator often declined to repeat measurements because of the unknown duration of the NTG response during each measurement, since it appeared to vary with different conditions. Furthermore, concerns about NTG-related BJR hindered repeated intracoronary NTG administration. Second, the single-center registry limited the external validity of the findings. A larger multicenter registry study should be conducted in the future. Lastly, owing to the small sample size and the rarity of the NTG-related complication, the details regarding the safe standardization of this approach across different clinical settings were limited.

## 5. Conclusions

This study showed a close correlation between NTG-Pd/Pa and FFR measurements under various conditions. The optimal cutoff value of NTG-Pd/Pa for predicting FFR ≤ 0.8 was reliable and has been validated. The diagnostic performance of NTG-Pd/Pa may be better than that of RFR in predicting FFR ≤ 0.8. However, the clinical benefits of NTG-Pd/Pa-guided revascularization are lacking, and a randomized controlled trial is required to evaluate the efficacy of NTG-Pd/Pa. Furthermore, NTG dose-response studies should be considered in the future.

## Data Availability

The original contributions presented in the study are included in the article/Supplementary Materials, and further inquiries can be directed to the corresponding author, C.-B.J.

## Supplementary Materials

The following supporting information can be downloaded at: https://www.mdpi.com/article/10.3390/jcm13226716/s1, Supplementary Material Figure S1: Workflow of the study protocol (proctored cohort); Supplementary Figure S2: Workflow of the study protocol (non-proctored cohort); Supplementary Figure S3: Acquisition of NTG-Pd/Pa values; Supplementary Figure S4: Receiver-operating characteristic curve of NTG-Pd/Pa for predicting a positive FFR in the pooled cohort and cohorts receiving different dosages of intracoronary NTG; Supplementary Table S1: Baseline characteristics, biochemical profiles, medications, and target vessel characteristics of different cohorts; Supplementary Table S2: Literature sources of the diagnostic performance of different physiological indices in predicting FFR ≤ 0.8.

## References

[1] Neumann F.J., Sousa-Uva M., Ahlsson A., Alfonso F., Banning A.P., Benedetto U., Byrne R.A., Collet J.P., Falk V., Head S.J. (2019). 2018 ESC/EACTS Guidelines on myocardial revascularization. Eur. Heart J..

[2] Ueng K.C., Chiang C.E., Chao T.H., Wu Y.W., Lee W.L., Li Y.H., Ting K.H., Su C.H., Lin H.J., Su T.C. (2023). 2023 Guidelines of the Taiwan Society of Cardiology on the diagnosis and management of chronic coronary syndrome. Acta Cardiol. Sin..

[3] Gotberg M., Cook C.M., Sen S., Nijjer S., Escaned J., Davies J.E. (2017). The evolving future of instantaneous wave-free ratio and fractional flow reserve. J. Am. Coll. Cardiol..

[4] Davies J.E., Sen S., Dehbi H.M., Al-Lamee R., Petraco R., Nijjer S.S., Bhindi R., Lehman S.J., Walters D., Sapontis J. (2017). Use of the instantaneous wave-free ratio or fractional flow reserve in PCI. N. Engl. J. Med..

[5] Gotberg M., Christiansen E.H., Gudmundsdottir I.J., Sandhall L., Danielewicz M., Jakobsen L., Olsson S.E., Ohagen P., Olsson H., Omerovic E. (2017). Instantaneous wave-free ratio versus fractional flow reserve to guide PCI. N. Engl. J. Med..

[6] Bernstein L., Friesinger G.C., Lichtlen P.R., Ross R.S. (1966). The effect of nitroglycerin on the systemic and coronary circulation in man and dogs: Myocardial blood flow measured with xenon. Circulation.

[7] Martin-Reyes R., de la Torre Hernandez J.M., Franco-Pelaez J., Lopez-Palop R., Telleria Arrieta M., Amat Santos I.J., Carrillo Saez P., Sanchez-Recalde A., Sanmartin Pena J.C., Garcia Camarero T. (2016). The use of the acute Pd/Pa drop after intracoronary nitroglycerin infusion to rule out significant FFR: CANICA (Can intracoronary nitroglycerin predict fractional flow reserve without adenosine?) multicenter study. Catheter. Cardiovasc. Interv..

[8] Shah S.P., Waxman S. (2013). Two cases of Bezold-Jarisch reflex induced by intra-arterial nitroglycerin in critical left main coronary artery stenosis. Tex. Heart Inst. J..

[9] De Bruyne B., Pijls N.H., Kalesan B., Barbato E., Tonino P.A., Piroth Z., Jagic N., Mobius-Winkler S., Rioufol G., Witt N. (2012). Fractional flow reserve-guided PCI versus medical therapy in stable coronary disease. N. Engl. J. Med..

[10] Jong C.B., Liao M.T., Lu T.S., Meng S.W., Chen C.K., Tsai Y.C., Kuo J.C., Wu C.C. (2022). Efficacy and safety of high-dose intracoronary adenosine injection in fractional flow reserve assessment. Acta Cardiol. Sin..

[11] Jong C.B., Lu T.S., Lin L., Chen T.Y., Liao M.T., Kuo J.C. (2024). Effect of prolonged pressure equalization on final drifting during pressure wire studies. Sci. Rep..

[12] Svanerud J., Ahn J.M., Jeremias A., van’t Veer M., Gore A., Maehara A., Crowley A., Pijls N.H.J., De Bruyne B., Johnson N.P. (2018). Validation of a novel non-hyperaemic index of coronary artery stenosis severity: The Resting Full-cycle Ratio (VALIDATE RFR) study. EuroIntervention.

[13] Youden W.J. (1950). Index for rating diagnostic tests. Cancer.

[14] Leisenring W., Alonzo T., Pepe M.S. (2000). Comparisons of predictive values of binary medical diagnostic tests for paired designs. Biometrics.

[15] DeLong E.R., DeLong D.M., Clarke-Pearson D.L. (1988). Comparing the areas under two or more correlated receiver operating characteristic curves: A nonparametric approach. Biometrics.

[16] Melenovsky V., Wichterle D., Malik J., Simek J., Hradec J., Ceska R., Malik M. (2002). Nitroglycerin induced syncope occurs in subjects with delayed phase shift of baroreflex action. Pacing Clin. Electrophysiol..

[17] Come P.C., Pitt B. (1976). Nitroglycerin-induced severe hypotension and bradycardia in patients with acute myocardial infarction. Circulation.

[18] Egashira K., Inou T., Hirooka Y., Yamada A., Urabe Y., Takeshita A. (1993). Evidence of impaired endothelium-dependent coronary vasodilatation in patients with angina pectoris and normal coronary angiograms. N. Engl. J. Med..

[19] Habazettl H., Vollmar B., Christ M., Baier H., Conzen P.F., Peter K. (1994). Heterogeneous microvascular coronary vasodilation by adenosine and nitroglycerin in dogs. J. Appl. Physiol..

[20] Asrress K.N., Williams R., Lockie T., Khawaja M.Z., De Silva K., Lumley M., Patterson T., Arri S., Ihsan S., Ellis H. (2017). Physiology of angina and its alleviation with nitroglycerin: Insights from invasive catheter laboratory measurements during exercise. Circulation.

[21] Patterson T., Rivolo S., Burkhoff D., Schreuder J., Briceno N., Allen C., Williams R., Arri S., Asrress K.N., Joseph J. (2021). Physiological impact of afterload reduction on cardiac mechanics and coronary hemodynamics following isosorbide dinitrate administration in ischemic heart disease. J. Cardiovasc. Transl. Res..

[22] Kumar G., Desai R., Gore A., Rahim H., Maehara A., Matsumura M., Kirtane A., Jeremias A., Ali Z. (2020). Real world validation of the nonhyperemic index of coronary artery stenosis severity-Resting full-cycle ratio-RE-VALIDATE. Catheter. Cardiovasc. Interv..

[23] Lee J.M., Choi K.H., Park J., Hwang D., Rhee T.M., Kim J., Park J., Kim H.Y., Jung H.W., Cho Y.K. (2019). Physiological and clinical assessment of resting physiological indexes. Circulation.

[24] Sen S., Escaned J., Malik I.S., Mikhail G.W., Foale R.A., Mila R., Tarkin J., Petraco R., Broyd C., Jabbour R. (2012). Development and validation of a new adenosine-independent index of stenosis severity from coronary wave-intensity analysis: Results of the ADVISE (ADenosine Vasodilator Independent Stenosis Evaluation) study. J. Am. Coll. Cardiol..

[25] Escaned J., Echavarría-Pinto M., Garcia-Garcia H.M., van de Hoef T.P., de Vries T., Kaul P., Raveendran G., Altman J.D., Kurz H.I., Brechtken J. (2015). Prospective assessment of the diagnostic accuracy of instantaneous wave-free ratio to assess coronary stenosis relevance: Results of ADVISE II International, Multicenter Study (Adenosine Vasodilator Independent Stenosis Evaluation II). Cardiovasc. Interv..

[26] Goto R., Takashima H., Ohashi H., Ando H., Suzuki A., Sakurai S., Nakano Y., Sawada H., Fujimoto M., Suzuki Y. (2021). Independent predictors of discordance between the resting full-cycle ratio and fractional flow reserve. Heart Vessel..

[27] Malmberg S., Lauermann J., Karlström P., Gulin D., Barmano N. (2023). Resting full-cycle ratio versus fractional flow reserve: A SWEDEHEART-registry-based comparison of two physiological indexes for assessing coronary stenosis severity. J. Interv. Cardiol..

[28] Kato Y., Dohi T., Chikata Y., Fukase T., Takeuchi M., Takahashi N., Endo H., Nishiyama H., Doi S., Okai I. (2021). Predictors of discordance between fractional flow reserve and resting full-cycle ratio in patients with coronary artery disease: Evidence from clinical practice. J. Cardiol..

[29] Johnson N.P., Jeremias A., Zimmermann F.M., Adjedj J., Witt N., Hennigan B., Koo B.K., Maehara A., Matsumura M., Barbato E. (2016). Continuum of vasodilator stress from rest to contrast medium to adenosine hyperemia for fractional flow reserve assessment. Cardiovasc. Interv..

[30] Jong C.-B., Kuo J.-C., Lin I.C. (2024). Kidney protection strategy lowers the risk of contrast-associated acute kidney injury. PLoS ONE.

