# Supplementary material for: Comparison of Nitroglycerin-Induced Pressure Ratio Drop and Resting Full-Cycle Ratio in a Pressure Wire Study
Source: J Clin Med. 2024 Nov 8;13(22):6716. doi: 10.3390/jcm13226716 (PMC11594636; doi:10.3390/jcm13226716)
Supplement: Supplementary file 1 [file jcm-13-06716-s001.zip › jcm-3237160-supplementary.pdf]

Supplementary Materials

Supplementary Figures

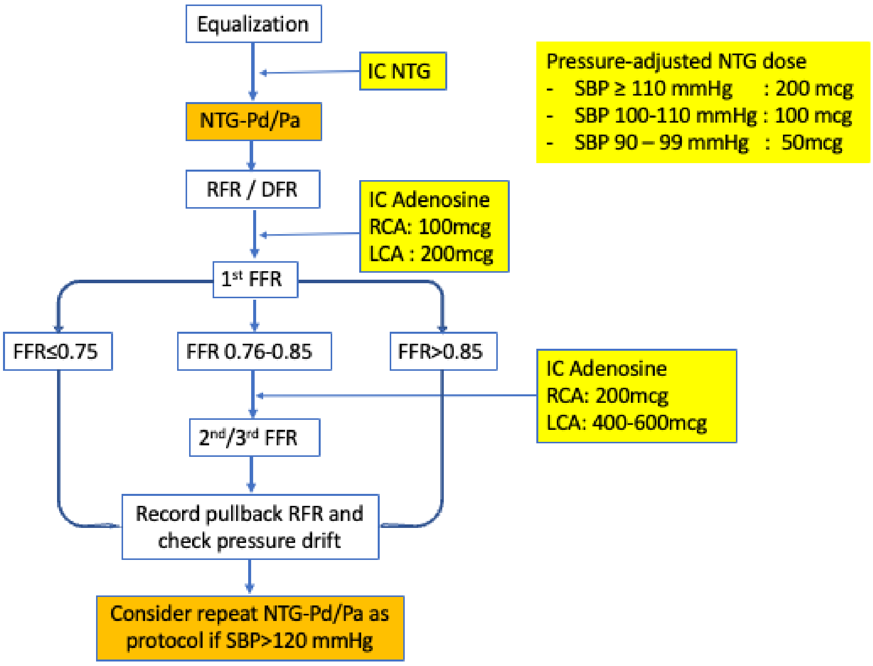

Supplementary Figure S1. Workflow of the study protocol (proctored cohort)

The dosage of intracoronary NTG administered during NTG-Pd/Pa measurement is adjusted according to the instant blood pressure. The RFR and DFR are measured after the Pd/Pa ratio is stabilized. The FFR value is obtained after inducing hyperemia with intracoronary adenosine. The NTG-Pd/Pa measurements are repeated at the operator's discretion after checking for pressure drift. DFR, diastolic hyperemia-free ratio; IC, intracoronary; LCA, left coronary artery; NTG, nitroglycerin; NTG-Pd/Pa, nitroglycerin-induced acute drop in Pd/Pa; Pa, aortic pressure; Pd, distal coronary pressure; RCA, right coronary artery; RFR, resting full-cycle ratio; SBP, systolic blood pressure.

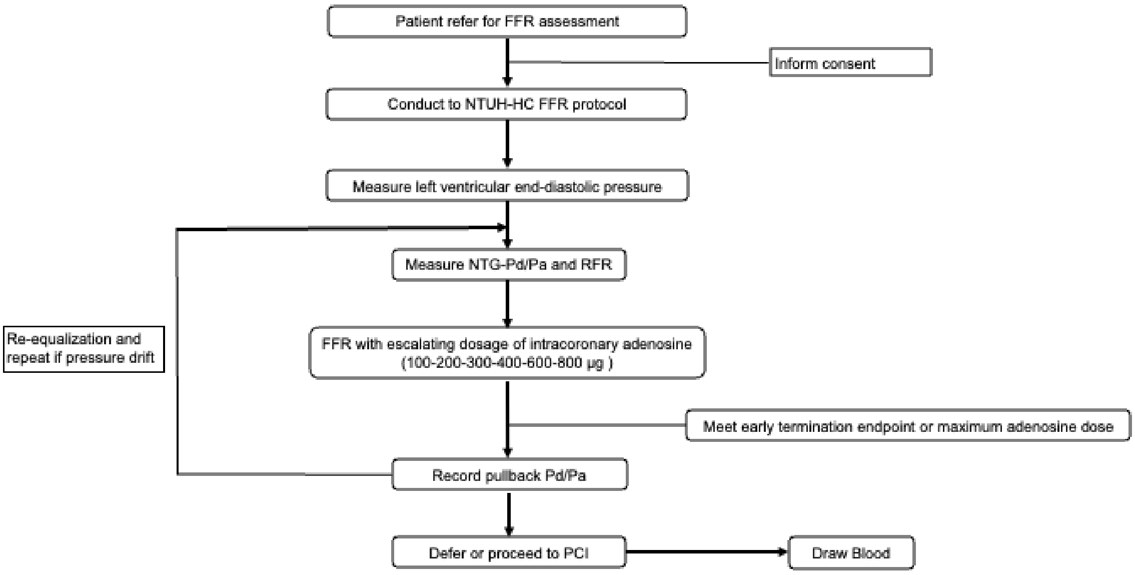

Supplementary Figure S2. Workflow of study protocol (non-proctored cohort)

The doses of intracoronary adenosine were gradually increased from 100 μg to 200, 300, 400, 600, and 800 μg during the FFR assessment.

Abbreviation: FFR, fractional flow reserve; NTG-Pd/Pa, nitroglycerine-induced acute drop in Pd/Pa; National Taiwan University Hospital Hsin-Chu Branch; PCI, percutaneous coronary intervention; RFR, resting full-cycle ratio.

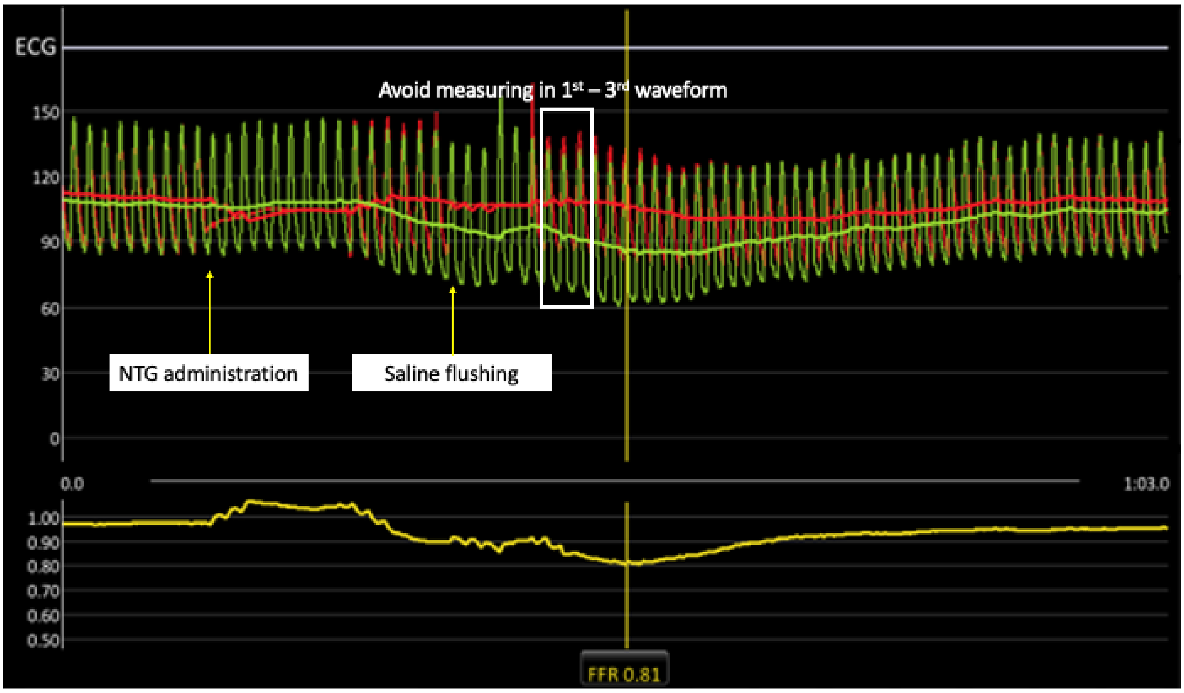

Supplementary Figure S3. Acquisition of NTG-Pd/Pa values

Intracoronary NTG administration induces an acute decrease in Pd/Pa. The NTG-Pd/Pa value is acquired at the lowest Pd/Pa, and measurements of the first three pressure waveforms are avoided after the initiation of the aortic pressure signal (white frame). NTG, nitroglycerin; NTG-Pd/Pa, nitroglycerin-induced acute drop in Pd/Pa; Pa, aortic pressure; Pd, distal coronary pressure.

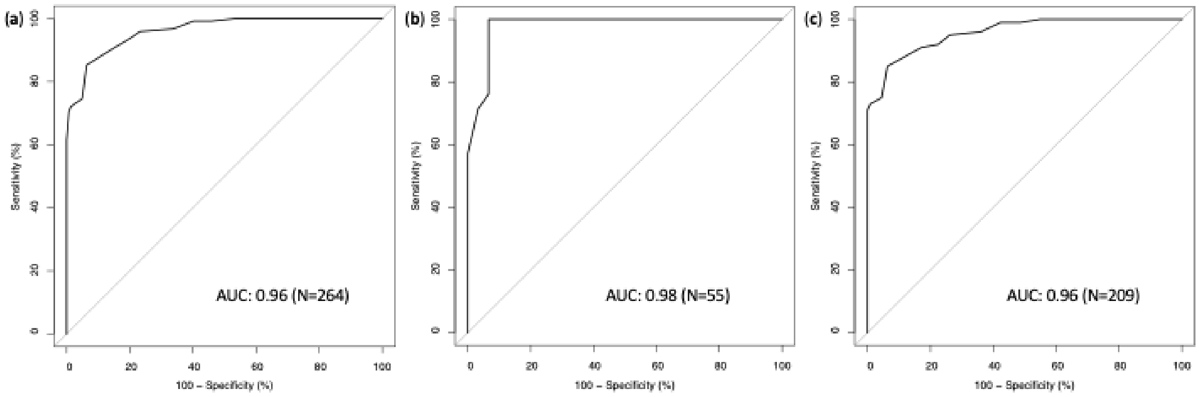

Supplementary Figure S4 Receiver-operating characteristic curve of NTG-Pd/Pa for predicting a positive FFR in the pooled cohort and cohorts receiving different intracoronary NTG doses

The AUC of NTG-Pd/Pa for predicting a positive FFR is high (all AUC>0.95) in the pooled cohort (**a**), vessels that received 100 µg (**b**), and vessels the received 200 µg (**c**). AUC, Area under the ROC curve; FFR, fractional flow reserve; NTG, nitroglycerin; NTG-Pd/Pa, nitroglycerin-induced acute drop in Pd/Pa; ROC, receiver-operating characteristic; Pa, aortic pressure; Pd, distal coronary pressure

Supplementary Tables

Supplementary Table S1. Baseline characteristics, biochemical profiles, medications, and target vessel characteristics of different cohorts

	Total	Proctored	Non-proctored
Patients	N = 125	N = 44	N = 81
Age (years)	66.5 ± 11.0	65.4 ± 10.9	67.2 ± 11.1
Sex (male)	97 (77.6%)	36 (81.8%)	61 (75.3%)
Body mass index	25.8 ± 4.4	24.9 ± 3.7	26.3 ± 4.6
Current smoker	19 (15.2%)	11 (25.0%)	8 (9.9%)
Hypertension	90 (72.0%)	31 (70.5%)	59 (72.8%)
Diabetes mellitus	60 (48.0%)	22 (50%)	38 (46.9%)
Hyperlipidaemia	95 (76.0%)	37 (84.1%)	58 (71.6%)
Heart failure	25 (20.0%)	9 (20.5%)	16 (19.8%)
Previous myocardial infarction	26 (20.8%)	7 (15.9%)	19 (23.5%)
Chronic kidney disease[a]	48 (38.4%)	21 (47.7%)	27 (33.3%)
<u>Clinical presentation</u>			
Acute coronary syndrome	27 (21.6%)	7 (15.9%)	20 (24.7%)
Chronic coronary syndrome	80 (64.0%)	27 (61.4%)	53 (65.4%)
Heart failure and others	18 (14.4%)	10 (22.7%)	8 (9.9%)
<u>Extent of coronary artery disease</u>			
Left main disease	9 (7.2%)	4 (9.1%)	5 (6.2%)
Multiple vessel disease	87 (69.6%)	32 (72.7%)	55 (67.9%)
<u>Laboratory data</u>			

Estimated GFR[b]	69.4 ± 23.3	65.3 ± 25.6	71.6 ± 22.0
Hemoglobin (median)	14.3 (12.4, 15.3)	14.4 (11.1, 15.3)	14.3 (12.7, 15.4)
Vessel	N = 202	N = 80	N = 122
Left anterior descending artery	96 (47.5%)	34 (42.5%)	62 (50.8%)
Left circumflex artery	47 (23.3%)	22 (27.5%)	25 (20.5%)
Right coronary artery	44 (21.8%)	14 (17.5%)	30 (24.6%)
Ramus intermediate or Diagonal branch	15 (7.4%)	10 (12.5%)	5 (4.1%)
Lesion location			
Ostium or proximal	65 (32.2%)	25 (31.3%)	40 (33.1%)
Middle	67 (33.2%)	24 (30.0%)	43 (35.5%)
Distal	40 (19.8%)	17 (21.3%)	23 (19.0%)
Major branches	29 (14.4%)	14 (17.5%)	15 (12.4%)
Diameter stenosis			
<50 %	20 (9.9%)	8 (10.0%)	12 (9.8%)
50%–70 %	170 (84.2%)	66 (82.5%)	104 (85.2%)
>70 %	12 (5.9%)	6 (7.5%)	6 (4.9%)
Physiological indexes at pre-intervention			
FFR≤0.8	100 (50.5%)	43 (55.8%)	57 (47.1%)
FFR 0.81–0.9	57 (28.8%)	18 (23.4%)	39 (32.2%)
FFR>0.9	41 (20.7%)	16 (20.8%)	25 (20.7%)
Median FFR	0.80	0.79	0.82

	(0.74, 0.89)	(0.69, 0.89)	(0.75, 0.89)
Median RFR	0.92	0.91	0.92
	(0.85, 0.96)	(0.84, 0.95)	(0.88, 0.96)
Median NTG-Pd/Pa	0.86	0.85	0.87
	(0.80, 0.93)	(0.73, 0.92)	(0.81, 0.93)
Administered nitroglycerin doses			
100 μg	55 (20.8%)	16 (14.7%)	40 (25.5%)
200 μg	209 (79.2%)	93 (85.3%)	117 (74.5%)

[a]Estimated GFR ≤60 mL/min/1.73m^2

[b]Patients with end-stage renal disease were excluded.

Abbreviations: FFR: fractional flow reserve; GFR: glomerular filtration rate; NTG-Pd/Pa, nitroglycerin-induced acute drop in Pd/Pa; Pa, aortic pressure; Pd, distal coronary pressure; RFR, resting full-cycle ratio

Supplementary Table S2. Literature sources of the diagnostic performance of different physiological indices in predicting FFR≤0.8

	Study	Sample size	AUC	Cutoff	Accuracy	Sensiti-vity	Specific-ity
NTG-Pd/Pa	NTUH-HC	226	0.97	0.85	90.7	85.7	95.6
Contrast FFR	CONTRAST	763	0.93	0.83	85.8	75.8	95.3
RFR	NTUH-HC	226	0.91	0.89	83.6	74.1	93.0
iFR	ADVISE	690	0.90	0.89	82.5	73.0	87.8
RFR	VALIDATE	651	0.88	0.89	81.3	71.5	88.0
iFR	VALIDATE	651	0.88	0.89	80.8	69.3	88.8

AUC, area under the curve; FFR, fractional flow reserve; GFR, glomerular filtration rate; iFR, instantaneous wave-free ratio; NTG-Pd/Pa, nitroglycerin-induced acute drop in Pd/Pa; Pa, aortic pressure; Pd, distal coronary pressure; RFR, resting full-cycle ratio
